# Use of Cyclosporine Therapy in Steroid Resistant Nephrotic Syndrome (SRNS): A Review

**DOI:** 10.5539/gjhs.v8n4p136

**Published:** 2015-08-06

**Authors:** Syed Raza Shah, Areeba Altaf, Mohammad Hussham Arshad, Anum Mari, Sahir Noorani, Eraj Saeed, Areesh Amir Mevawalla, Zaiyn Ul Haq, Muhammad Ehsan Faquih

**Affiliations:** 1 Department of Medicine, Dow University of Health Sciences (DUHS), Karachi, Pakistan; 2 Department of Medicine, Aga Khan University of Health Sciences, Karachi, Pakistan; 3 Department of Biological Sciences, The Lyceum, Karachi, Pakistan; 4 Department of Biological Sciences, Karachi Grammar School, Karachi, Pakistan

## Abstract

A chronic, progressive disorder Steroid Resistant Nephrotic Syndrome (SRNS) accounts for 10-20% of all children with Nephrotic Syndrome. It is a heterogeneous disorder comprised of persistent edema, proteinuria, hypoalbuminemia and hyperlipidemia. Treatment for steroid-resistant nephrotic syndrome (SRNS) is challenging and children who suffer from SRNS require aggressive treatment to achieve remission. Calcineurin inhibitors have been used more in an empirical manner than on the basis of clear rationale. It was in 1984 when cyclosporine was first considered for the treatment of steroid resistant nephrotic syndrome. Cyclosporin is a calcineurin inhibitor that suppresses immune response by downregulating the transcription of various cytokine genes. Till now many studies have been conducted to determine dosages, duration of therapy, side effects and advantages of cyclosporine. Treatment of SRNS remains a difficult challenge in pediatric nephrology. Treatment should be individualized according to the underlying histopathology, and clinical and environmental conditions of the children. There is an urgent need to distinguish as soon as possible those patients who may benefit from prolonged immunosuppressive treatment from those who will not benefit from such treatment and who will just suffer from its major side effects. The emerging evidence that the majority of genetic forms of SRNS should receive symptomatic treatment only, should also be clinically tested and studies baring its significance should be evaluated in the future.

## 1. Background and Introduction

A chronic, progressive disorder Steroid Resistant Nephrotic Syndrome (SRNS) accounts for around 10 to 20% of all children with Nephrotic Syndrome (Banerjee, 2002). It is a heterogeneous disorder comprised of persistent edema, proteinuria, hypoalbuminemia and hyperlipidemia. Various studies have shown that over 80% of children with initial episode of Nephrotic Syndrome respond well to steroids but almost 10-20% do not respond to steroids and are known to have SRNS for which therapeutics other than steroids are used as a treatment. (Bhimma, 2005) Treatment primarily includes steroids but when there is no remission, potent immunosuppressants are used. It has been observed that certain factors have known to contribute to steroid resistance which include hypertension, hematuria, hypertension plus hematuria, proteinuria(>10 g/day), elevated plasma creatinine, black race, presenting in infancy, renal biopsy showing Tubulointerstitial disease, selectivity index >0.2 and tubular proteinuria (Banerjee, 2002; Bhimma, 2005; Ramjee, Coovadia, & Adhikari, 1997). Unfortunatley patients with SRNS are highly prone to develop complications in comparison to children having steroid sensitive nephrotic syndrome. These complication include acute renal failure (White, Glasgow, & Mills, 1970), chronic renal failure, growth retardation, impaired immunity leading to infections like peritonitis and thrombosis (Bhimma, 2005).

## 2. Review and Discussion

Children suffering from nephrotic syndrome have shown to have minimal change glomerulonephritis (MCNS), focal segmental glomerulosclerosis (FSGS) or mesangial proliferative glomerulonephritis; hence most are suffering from Idiopathic Nephrotic Syndrome (Habib, 1993; Churg, Habib, & White, 1970).

SRNS and mainly FSGS have shown to have 50% risk of end stage renal disease within 5 years of diagnosis in case the patient does not manage to have partial or complete remission. Only 10-20% of nephrotic syndrome patients develop resistance to steroids but this fraction contributes disproportionately to end stage renal disease as compared to steroid sensitive nephrotic syndrome. If progressive renal impairment is controlled, complete or partial remission preserves renal function and brings about excellent long term results. However, End stage renal disease in patients with SRNS considerably reduces life expectancy, about 19 years after initiation of dialysis and around 40 years following transplantation (Kidney international Supplement, 2011; Gipson et al., 2006; Butani & Ramsamooj, 2009).

Among pediatric nephrologists there are two definitions of SRNS. The definition introduced by the International Study of Kidney Disease in Children (ISKDC) and used by the Arbeitsgemeinschaft für Pädiatrische Nephrologie (APN) is widely accepted and which states, ‘ No urinary remission within four weeks of prednisone therapy of 60 mg/m2/day’. The other definition, employed by the Society of French Speaking Pediatric Nephrologists says,’ No urinary remission following four weeks of prednisone at 60 mg/m2/day followed by three intravenous pulses of methylpredisolone’.(Brodehl, Krohn, & Ehrich, 1982; Niudet, 1994).

When SRNS is suspected, a meticulous search for the possibility of concurrent infection (e.g skin infection and sinusitis), compliance problem, drug interaction, or inappropriate dosage is necessary. If these secondary conditions are ruled out, tissue diagnosis from a renal biopsy is the next step. Histological findings of SRNS are, and rarely, secondary glomerulopathy such as amyloidosis is unexpectedly found. At the same time, analysis for mutational genes known to cause SRNS is recommended.

Few factors have to be evaluated when treating for SRNS. These include confirmation of resistance to steroids (usually oral prednisone or oral prednisolone), kidney biopsy to rule out secondary reasons of Nephrotic Syndrome, determine GFR at presentation due to long term risk of kidney failure and quantification of proteinuria to check treatment response(Kidney International Supplement, 2011).

Treatment for steroid-resistant nephrotic syndrome (SRNS) is challenging and children who suffer from SRNS require aggressive treatment to achieve remission. Thus, when intravenous high-dose methylprednisolone does not work, calcineurin inhibitors, such as cyclosporine, is used as the first line of treatment (Tejani & Ingulli, 1995). Cyclosporine has shown to have higher rate of remission as compared to other immunosuppressant therapies used for the treatment of SRNS (Tahar & Rachid, 2010). Calcineurin inhibitors have been used more in an empirical manner than on the basis of clear rationale (Tejani & Ingulli, 1995). It was in 1984 when cyclosporine was first considered for the treatment of SRNS. Till now many studies have been conducted to determine dosages, duration of therapy, side effects and advantages of cyclosporine (Adhikari & Coovadia, 1994).

Cyclosporin is a calcineurin inhibitor that suppresses immune response by downregulating the transcription of multiple cytokine genes. The most significant of these cytokines is interleukin-2, which serves as the major activation factor for T cells in numerous immunologic processes. Cyclosporin inhibits cytokine production from T helper cells and also has an inhibitory effect on antigen presenting cells which are the main agents of T cell stimulation. A further effect of IL-2 inhibition is a reduction in B-cell activation and subsequent antibody production. IL-2 levels are known to become elevated during proteinuria and to normalize during remission in adults with idiopathic nephrotic syndrome and in children with Minimal change Nephrotic Syndrome (MCNS) or FSGS (Tejani & Ingulli, 1995). However, this pattern of interleukin-2 activity is felt to be part of a more widespread disorder of cellular immunity that results in nephrotic syndrome rather than being causal of proteinuria. It has been reported that cyclosporin has some antiproteinuric action on glomerular perm-selectivity to proteins that is unrelated to its immunosuppressive properties. Among these are an influence on perm-selectivity and charge selectivity and impairment of GFR. These data come from various human studies (Zietse et al., 1992; Meyrier, Noel, Auriche, & Callard, 1994; Ambalavanan et al., 1996; Heering et al., 2001) and animal models (Heering et al., 2001; Kokui et al., 1992, Schriiver et al., 1995; Desassis et al., 1997) with no immunologically mediated disease. Some studies revealed that lesions from the primary glomerular disease had either not regressed or had continued to progress (Meyrier, Noel, Auriche, & Callard, 1994; Ambalavanan et al., 1996; Chen, 2003).

Treatment of SRNS remains a difficult challenge in pediatric nephrology. At the moment there is no diagnostic marker for children displaying with nephrotic syndrome that can be used as a predictor of steroid resistance or responsiveness. The most important prognostic marker for children with nephrotic syndrome is their response to steroid treatment. However, Initial steroid treatment can be avoided only in patients with a family history of SRNS or in those who have a known gene mutation. According to a study, the predictors for Cyclosporin non-responsiveness were steroid resistance, non minimal change disease on biopsy and longer duration between onset of nephrotic syndrome and cyclosporin usage, irrespective of the age of onset of the disease (Iyengar, 2006)

Using cyclosporine therapy in SRNS has its consequences. Long-term Cyclosporin therapy in low doses is effective in the treatment of children with idiopathic Nephrotic syndrome (NS), but the rate of relapse is high after drug withdrawal (El-husseini, 2005). Treatment with cyclosporine has significant nephrotoxicity. This is a common effect of all calcineurin inhibitors; hence cyclosporine therapy needs regular monitoring. This can be achieved by measurement of cyclosporine blood concentration at *C*_0_ which is pre-dose concentration or *C*_2_ levels which is concentration at 2 h post-dose. Better assessment can be done by *C*_2_ measurement as *C*_0_ represents the trough blood levels which do not significantly represent function of cyclosporine intake. Studies have shown that this drug can be safely used in chidren with nephrotic syndrome and also in transplant patients. Moreover it has also been proved by yearly assessments of creatinine and creatine clearance that renal function remains stable for upto 20 years (Serkova & Christians, 2003; Midvedt et al., 2003; Fujinaga, 2006; Kandaswamy, 2007). According to recent researches once a day administration of the drug is more beneficial than the old twice a day administration due to former providing an absorption profile with peak blood concentration of cyclosporine which may cause remission of SRNS and prevent chronic cyclopsporine nephrotoxicity (Tanaka et al., 2004; Takeda, 2007).

According to a study, renal insufficiency developed in 6% and hypertension in 10% of patients (most patients with FSGS). (El-husseini, 2005) Furthermore, progression of the previous interstitial fibrosis and tubular atrophy was noted in two patients, suggesting a 17% incidence of cyclosporin nephrotoxicity. This analysis of the long-term risks of cyclosporin for childhood NS has identified two important findings: (Tejani & Ingulli, 1995) combined cyclosporin and alternate-day steroids can be highly effective in inducing complete remission in patients with SRNS and biopsy-proven IgM nephropathy, and (Tahar & Rachid, 2010) long-term use of cyclosporin in moderate doses with closely monitored levels can result in a relatively low incidence of nephrotoxicity.(Gregory et al., 1996) There was a higher incidence of cyclosporin dependence among young responders. Also, patients with cyclosporin resistance are at high risk for significant infections and CRF (Iyengar, 2006).

A number of trials have been done for cyclosporine as a treatment of SRNS. In Three Randomized Control Trials (RCTs) with 49 patients, 26 patients were given cyclosporine while 23 were given control therapy or placebo. It was concluded that complete remission resulted in 31% and partial remission in 38% was seen during a 6 month period of therapy. Similarly another trial which was conducted in 2011 in which cyclosporine was compared to mycophenolate added to high dose of dexamethasone for a 12 month durartion resulted in around 19% complete remission and around 26% partial remission (Garin et al., 1988; Lieberman & Tejani, 1996; Ponticelli et al., 1993; Gipson et al., 2011).

In the recent years, experts of the Indian Society of Pediatric Nephrology were involved in a 2-stage process to formulate guidelines, based on recent practices and available evidence, on management of SRNS children. Agreement of at least 80% participants formed an opinion (Gulati et al., 2009). The Expert Group emphasized that while all patients with SRNS should initially be referred to a pediatric nephrologist for evaluation, the subsequent care should be collaborative involving the primary pediatrician and the nephrologist. Following the diagnosis of SRNS (lack of remission despite treatment with prednisolone at 2 mg/kg/day for 4 weeks), all patients (with initial or late resistance) should undergo a kidney biopsy, before instituting specific and targeted treatment. Patients with idiopathic SRNS secondary to MCNS or FSGS should receive similar treatment. Effective therapy regimens include treatment with calcineurin inhibitors (Cyclosporine), combination of pulse corticosteroids with oral cyclophosphamide or intra-venous cyclophosphamide, tapering doses of alternate day corticosteroids. Supportive management comprises therapy with angiotensin converting enzyme inhibitors and statins. These guidelines are expected to enable standardization of care for patients with SRNS worldwide (Gulati et al., 2009).

KDIGO Clinical Practice Guideline for Glomerulonephritis published in 2012, suggested treatment recommendations for SRNS. These were calcineurin inhibitor (CNI) as initial therapy which should be continued for atleast 6 months and discontinued only if partial or complete remission of proteinuria is not observed; In case partial remission is achieved by 6 months then CNIs can be continued for minimum of 12 months. Other suggestions in these guidelines were low dose corticosteroid therapy with CNI therapy and ACE-I or ARBs were also recommended. In children who fail to respond to CNI therapy can be treated with mycophenolate mofetil, high-dose corticosteroids, or a combination of these agents in children who fail to achieve complete or partial remission with CNIs and corticosteroids. It was suggested to not to use cyclophosphamide in these children. These guidelines also mentioned treatment for patients with a relapse of nephrotic syndrome after complete remission, in which therapy should be restarted using oral corticosteroids, returning to previous successful immunosuppressive agent or an alternative immunosuppressive agent to minimize potential cumulative toxicity (Clinical Practice Guidelines KDIGO, 2012).

## 3. Conclusion

SRNS in children is a difficult disease with significant morbidity and mortality. However, remission is achievable with cyclosporine and other immunosuppressive agents. Although the prognosis of SRNS is complicated, an intensive treatment in the early stages of the disease may achieve remission in more than half of the patients. Thus, timely referral of pediatric SRNS patients to pediatric nephrology specialists for histological and genetic diagnosis and treatment is highly recommended. Treatment should be individualized according to the underlying histopathology, and clinical and environmental conditions of the children. (Kazi & Halawani, 2010) There is an urgent need to distinguish as soon as possible those patients who may benefit from prolonged immunosuppressive treatment from those who will not benefit from such treatment and who will just suffer from its major side effects. The emerging evidence that the majority of genetic forms of SRNS should receive symptomatic treatment only, should also be clinically tested and studies baring its significance should be evaluated in the future.

## References

[ref1] Adhikari M, Coovadia H. M (1994). Cyclosporin in steroidresistant nephrotic syndrome. SAMJ.

[ref2] Ambalavanan S, Fauvel J. P, Sibley R. K, Myers B. D (1996). Mechanism of the antiproteinuric effect of cyclosporine in membranous nephropathy. J Am Soc Nephrol.

[ref3] Banerjee S (2002). Steroid resistant nephrotic syndrome. The Indian Journal of Pediatrics.

[ref4] Bhimma R (2005). Steroid resistant nephrotic syndrome in children. Pediatric Oncall Journa.

[ref5] Brodehl J, Krohn H. P, Ehrich J. H (1982). The treatment of minimal change nephrotic syndrome (lipoid nephrosis): Cooperative studies of the Arbeitsgemeinschaft fur Padiatrische Nephrologie (APN. Klin Padiatr.

[ref6] Butani L, Ramsamooj R (2009). Experience with tacrolimus in children with steroid-resistant nephrotic syndrome. Pediatr Nephrol.

[ref7] Chen H, Tang Z, Zeng C, Hu W, Wang Q (2003). Pathological demography of native patients in a nephrology center in china. Chinese Medical Journal.

[ref8] Churg J, Habib R, White R. H (1970). Pathology of the nephrotic syndrome in children. Lancet.

[ref9] Desassis J. F, Raats C. J, Bakker M. A, Van, d B. J, Berden J. H (1997). Antiproteinuric effect of ciclosporin a in adriamycin nephropathy in rats. Nephron.

[ref10] El-Husseini A, El-Basuony F, Mahmoud I, Sheashaa H, Sabry A, Hassan R (2005). Long-term effects of cyclosporine in children with idiopathic nephrotic syndrome: a single-centre experience. Nephrology Dialysis Transplantation.

[ref11] Fujinaga S, Kaneko K, Muto T, Ohtomo Y, Murakami H, Yamashiro Y (2006). Independent risk factors for chronic cyclosporine induced nephropathy in children with nephrotic syndrome. Archives of Disease in Childhood.

[ref12] Garin EH, Orak JK, Hiott KL, Sutherland SE (1988). Cyclosporine therapy for steroid-resistant nephrotic syndrome. a controlled study. Am J Dis Child.

[ref13] Gipson D. S, Chin H, Presler T. P, Jennette C, Ferris M. E, Massengill S, Gibson K, Thomas D. B (2006). Differential risk of remission and ESRD in childhood FSGS. Pediatr Nephrol.

[ref14] Gipson D. S, Trachtman H, Kaskel F. J, Greene T. H, Radeva M. K, Gassman J. J (2011). Clinical trial of focal segmental glomerulosclerosis in children and young adults. Kidney International.

[ref15] Gregory M. J, Smoyer W. E, Sedman A, Kershaw D. B, Valentini R. P, Johnson K (1996). Long-term cyclosporine therapy for pediatric nephrotic syndrome: a clinical and histologic analysis. Journal of the American Society of Nephrology.

[ref16] Habib R (1993). A story of glomerulopathies: a pathologist's experience. Pediatr Nephrol.

[ref17] Heering P, Schneider A, Grabensee B, Plum J (2001). Effect of cyclosporin a on renal function in patients with glomerulonephritis. Dtsch Med Wochenschr.

[ref18] http://www.kdigo.org/clinical_practice_guidelines/pdf/KDIGO-GN-Guideline.pdf.

[ref19] Gulati A, Bagga A, Gulati S, Mehta K. P, Vijayakumar M, Indian Society of Pediatric Nephrology (2009). Management of steroid resistant nephrotic syndrome. Indian Pediatr.

[ref20] Iyengar A, Karthik S, Kumar A, Biswas S, Phadke K (2006). Cyclosporine in steroid dependent and resistant childhood nephrotic syndrome. Indian Pediatrics.

[ref21] Kandaswamy R, Humar A, Casingal V, Gillingham K. J, Ibrahim H, Matas A. J (2007). Stable kidney function in the second decade after kidney transplantation while on cyclosporine-based immunosuppression. Transplantation.

[ref22] Kari J. A, Halawani M (2010). Treatment of steroid resistant nephrotic syndrome in children. Saudi J Kidney Dis Transpl.

[ref23] Kokui K, Yoshikawa N, Nakamura H, Itoh H (1992). Cyclosporin reduces proteinuria in rats with aminonucleoside nephrosis. Journal of Pathology.

[ref24] Lieberman K. V, Tejani A (1996). A randomized double-blind placebo-controlled trial of cyclosporine in steroid-resistant idiopathic focal segmental glomerulosclerosis in children. Journal of the American Society of Nephrology.

[ref25] Meyrier A, Noel L. H, Auriche P, Callard P (1994). Long-term renal tolerance of cyclosporin a treatment in adult idiopathic nephrotic syndrome. Collaborative group of the societe de nephrology. Kidney Int.

[ref26] Midtvedt K, Fauchald P, Bergan S, H03ieggen A, Hallan S, Svarstad E (2003). C2 monitoring in maintenance renal transplant recipients: is it worthwhile?. Transplantation.

[ref27] Niaudet P (1994). Treatment of childhood steroid-resistant idiopathic nephrosis with a combination of cyclosporine and prednisone. French society of pediatric nephrology. J Pediatr.

[ref28] Ponticelli C, Rizzoni G, Edefonti A, Altieri P, Rivolta E, Rinaldi S (1993). A randomized trial of cyclosporine in steroid-resistant idiopathic nephrotic syndrome. Kidney International.

[ref29] Ramjee G, Coovadia H. M, Adhikari M (1997). Comparison of non-invasive methods of distinguishing steroid sensitive nephrotic syndrome from focal glomerulosclerosis. J Lab Clinic Med.

[ref30] Schrijver G, Assmann K. J, Wetzels J. F, Berden J. H (1995). Cyclosporin a reduces albuminuria in experimental anti-gbm nephritis independently from changes in gfr. Nephrology Dialysis Transplantation.

[ref31] Serkova N, Christians U (2003). Transplantation: toxicokinetics and mechanisms of toxicity of cyclosporine and macrolides. Current Opinion in Investigational Drugs.

[ref32] Steroid-resistant nephrotic syndrome in children (2011). Kidney Int Suppl.

[ref33] Tahar G, Rachid L. M (2010). Cyclosporine a and steroid therapy in childhood steroid-resistant nephrotic syndrome. Int J Nephrol Renovasc Dis.

[ref34] Takeda A, Horike K, Onoda H, Ohtsuka Y, Yoshida A, Uchida K, Morozumi K (2007). Benefits of cyclosporine absorption profiling in nephrotic syndrome: preprandial once-daily administration of cyclosporine microemulsion improves slow absorption and can standardize the absorption profile. Nephrology (Carlton).

[ref35] Tanaka H, Nakahata T, Ito E (2004). Single-dose daily administration of cyclosporin02a for relapsing nephrotic syndrome. Pediatric Nephrology.

[ref36] Tejani A, Ingulli E (1995). Current concepts of pathogenesis of nephrotic syndrome. Contrib Nephrol.

[ref37] White R. H. R, Glasgow E. F, Mills R. J (1970). Clinicopathological studies of nephrotic syndrome in childhood. Lancet.

[ref38] Zietse R, Wenting G. J, Kramer P, Schalekamp M. A, Weimar W (1992). Effects of cyclosporin a on glomerular barrier function in the nephrotic syndrome. Clin Sci (Lond).

